# Evaluation of left ventricular filling pressure by echocardiography in patients with atrial fibrillation

**DOI:** 10.1186/s44156-024-00048-x

**Published:** 2024-06-03

**Authors:** Faraz H. Khan, Debbie Zhao, Jong-Won Ha, Sherif F. Nagueh, Jens-Uwe Voigt, Allan L. Klein, Einar Gude, Kaspar Broch, Nicholas Chan, Gina M. Quill, Robert N. Doughty, Alistair Young, Ji-Won Seo, Eusebio García-Izquierdo, Vanessa Moñivas-Palomero, Susana Mingo-Santos, Tom Kai Ming Wang, Stephanie Bezy, Nobuyuki Ohte, Helge Skulstad, Carmen C. Beladan, Bogdan A. Popescu, Shohei Kikuchi, Vasileios Panis, Erwan Donal, Espen W. Remme, Martyn P. Nash, Otto A. Smiseth

**Affiliations:** 1 Institute for Surgical Research, Division of Cardiovascular and Pulmonary Diseases, Oslo University Hospital, University of Oslo, Rikshospitalet, Oslo, N-0027 Norway; 2 Auckland Bioengineering Institute, University of Auckland, Auckland, New Zealand; 3 Cardiology Division, Severance Hospital, Yonsei University College of Medicine, Seoul, Korea; 4 Methodist DeBakey Heart and Vascular Center, Houston, TX USA; 5 Department of Cardiovascular Diseases, Department of Cardiovascular Sciences, University Hospitals Leuven, Catholic University of Leuven, Leuven, Belgium; 6 Cleveland Clinic, Cleveland, OH USA; 7 Department of Medicine, University of Auckland, Auckland, New Zealand; 8 Cardiology Unit, Hospital Universitario Puerta de Hierro Majadahonda, Madrid, Spain; 9 Department of cardiology, Nagoya City University Graduate School of Medical Sciences, Nagoya, Japan; 10 University of Medicine and Pharmacy “Carol Davila”, Emergency Institute for, Cardiovascular Diseases “Prof. Dr. C. C. Iliescu”, Sos. Fundeni 258, sector 2, Euroecolab, Bucharest, 0223228 Romania; 11 Department of Cardiology, CHU Rennes and Inserm, LTSI, University of Rennes, Rennes, France; 12 The Intervention Centre, Oslo University Hospital, Rikshospitalet, Oslo Norway

**Keywords:** Atrial fibrillation, Diastolic function, Echocardiography, Left atrium, Left ventricle, Filling pressure

## Abstract

**Background:**

Echocardiography is widely used to evaluate left ventricular (LV) diastolic function in patients suspected of heart failure. For patients in sinus rhythm, a combination of several echocardiographic parameters can differentiate between normal and elevated LV filling pressure with good accuracy. However, there is no established echocardiographic approach for the evaluation of LV filling pressure in patients with atrial fibrillation. The objective of the present study was to determine if a combination of several echocardiographic and clinical parameters may be used to evaluate LV filling pressure in patients with atrial fibrillation.

**Results:**

In a multicentre study of 148 atrial fibrillation patients, several echocardiographic parameters were tested against invasively measured LV filling pressure as the reference method. No single parameter had sufficiently strong association with LV filling pressure to be recommended for clinical use. Based on univariate regression analysis in the present study, and evidence from existing literature, we developed a two-step algorithm for differentiation between normal and elevated LV filling pressure, defining values ≥ 15 mmHg as elevated. The parameters in the first step included the ratio between mitral early flow velocity and septal mitral annular velocity (septal E/e’), mitral E velocity, deceleration time of E, and peak tricuspid regurgitation velocity. Patients who could not be classified in the first step were tested in a second step by applying supplementary parameters, which included left atrial reservoir strain, pulmonary venous systolic/diastolic velocity ratio, and body mass index. This two-step algorithm classified patients as having either normal or elevated LV filling pressure with 75% accuracy and with 85% feasibility. Accuracy in EF ≥ 50% and EF < 50% was similar (75% and 76%).

**Conclusions:**

In patients with atrial fibrillation, no single echocardiographic parameter was sufficiently reliable to be used clinically to identify elevated LV filling pressure. An algorithm that combined several echocardiographic parameters and body mass index, however, was able to classify patients as having normal or elevated LV filling pressure with moderate accuracy and high feasibility.

## Background

Echocardiography is widely used in the evaluation of patients suspected of heart failure (HF) and includes assessment of left ventricular (LV) diastolic function. As suggested by consensus documents from the European Association of Cardiovascular Imaging (EACVI) and the American Society of Echocardiography (ASE), algorithms that combine several echocardiographic parameters can differentiate between normal and elevated LV filling pressure with good accuracy in both heart failure with reduced ejection fraction and heart failure with preserved ejection fraction [1, 2]. The validity of the proposed algorithms was confirmed in studies that used invasively measured LV filling pressure as the gold standard [3, 4]. These algorithms, however, are not recommended in patients with atrial fibrillation as some of the indices have only a weak association with LV filling pressure in the presence of atrial fibrillation. This is a significant limitation of current diagnostic methods since atrial fibrillation occurs in 25–65% of heart failure patients, with the highest prevalence in heart failure with preserved ejection fraction and in the elderly [5, 6].

Several different echocardiographic parameters have been proposed as markers of LV filling pressure in atrial fibrillation, but their validation is limited, and they have not been adopted in clinical routine [1, 2]. The objective of the present study was to determine if a combination of multiple echocardiographic parameters may be used to more effectively evaluate LV filling pressure in atrial fibrillation. In addition, since clinical condition and history might provide added information, we tested if using a combination of echocardiographic and clinical parameters could improve the accuracy of LV filling pressure assessment.

## Methods

### Data collection

This multicentre study was conducted at Oslo University Hospital, Rikshospitalet (Oslo, Norway), Auckland City Hospital (Auckland, New Zealand), Methodist DeBakey Heart and Vascular Centre (Houston, TX, USA), Severance Hospital, Yonsei University College of Medicine (Seoul, South Korea), Cleveland Clinic (Cleveland, OH, USA), Hospital Universitario Puerta de Hierro, Majadahonda (Madrid, Spain), Nagoya City University, Graduate School of Medical Sciences (Nagoya, Japan), Emergency Institute for Cardiovascular Diseases “Prof. Dr. C. C. Iliescu” (Bucharest, Romania), University Hospital Leuven (Leuven, Belgium) and Department of Cardiology, CHU Rennes and Inserm, LTSI, University of Rennes, Rennes, France.

A total of 148 patients with suspected heart failure or other indication for cardiac catheterisation with persistent or chronic atrial fibrillation referred for right- or left-sided heart catheterisation were included (77 prospectively and 71 retrospectively). Patients with complex congenital heart disease, cardiac transplants, end-stage liver disease, mitral stenosis or mitral annular calcification resulting in significant mitral stenosis, prosthetic mitral valve, severe aortic stenosis, severe mitral or tricuspid regurgitation, and atrial fibrillation with rapid average ventricular rate at rest (> 120 bpm), were excluded.

The study was approved by the Regional Ethics Committee and Institutional Review Boards at each participating centre.

### Clinical data

Age, sex, height, weight, blood pressure, and heart rate, were obtained for all patients, with the latter two acquired during both echocardiography and cardiac catheterisation to ensure there were no significant differences. In addition, data on history of hypertension, diabetes and blood pressure lowering medication were obtained. NT-proBNP was measured in 107 (72%) patients.

### Echocardiographic imaging

All echocardiographic recordings were obtained and analysed by experienced investigators without knowledge of the invasive haemodynamic data.

Recordings were obtained with a minimum of 5 consecutive heart beats for all measurements. For each variable, the average value was used in our analysis. Echocardiography was performed either simultaneously with, or within 8 h of haemodynamic assessment. Cycles with the shortest R-R intervals where mitral inflow ended in LV systole, were excluded. All measurements were performed according to current recommendations [1, 7].

Being a multicentre, multivendor study, different echocardiography equipment was used at the participating centres, including GE Healthcare (Echopac), Philips (QLAB), and Siemens (VVI, TOMTEC).

A standard transthoracic echocardiography examination was performed including left atrial (LA) strain, LA volumes, LV ejection fraction (EF), as well as LA and LV dimensions and LV mass. Peak mitral early (E) velocity, peak septal and lateral mitral annular velocities (e′), peak pulmonary venous (PV) systolic (S) and diastolic (D) velocities and their ratio, and peak tricuspid regurgitation (TR) velocity were measured. Saline contrast or ultrasound enhancing agents were not routinely administered in cases with an incomplete TR jet. Strain values were measured by speckle tracking echocardiography using frame rates from 40 to 80/s, according to consensus documents [8, 9]. Left atrial reservoir strain was measured on images acquired either in apical 4-chamber view or in biplane. For measurement of LA strain some centres (*n* = 5) used software which was developed primarily for measuring LV strain while others (*n* = 4) used software dedicated for measuring LA strain.

### Cardiac catheterisation

Left ventricular filling pressure was measured during diagnostic right heart catheterisation as mean pulmonary capillary wedge pressure (PCWP) in 66 patients, and during diagnostic left heart catheterisation as LV end-diastolic pressure (LVEDP) in 47 patients. Pulmonary capillary wedge position was verified by fluoroscopic guidance and from the pressure waveform. In 35 patients, LV filling pressure was measured in the LA as mean LA pressure after transeptal catheterisation. Left ventricular filling pressure (measured as either PCWP, mean LA pressure, or LVEDP) ≥ 15 mmHg was considered elevated. All invasive measurements were averaged over 5 beats and obtained during end-expiration.

### Analyses

Univariate linear regression analysis was used to assess the correlation between clinical or echocardiographic variables and LV filling pressure. In addition, we investigated how an algorithm that combined several echocardiographic and clinical parameters could be used to differentiate between normal and elevated LV filling pressure. The selection of parameters was based in part on previous studies in patients with sinus rhythm as summarised in current guidelines [1, 2], on previous observations in single parameter studies of atrial fibrillation [10–15], and on results from analysis of single parameters that exhibited statistically significant linear relationships to LV filling pressure in the present study.

A two-tailed *p* < 0.05 was considered significant for all statistical analyses, and the Pearson correlation coefficient (r-value) was computed to assess the strength of the linear relationships. All statistical analyses were performed using either Graphpad prism version 9.5.0 or SPSS software version 24.0.

The feasibility of application of the algorithm was calculated as the percentage of patients with a sufficient number of measured parameters to produce a classification, while accuracy was calculated as the percentage of patients correctly classified as having either normal or elevated LV filling pressure.

## Results

Table 1 summarises key clinical and haemodynamic characteristics in the patient population.

**Table 1:** Clinical and haemodynamic characteristics

Variables	Median (interquartile range) or %
*Clinical variables*	
Age, years	68 (62–75)
Female	26%
Body mass index, kg/m^2^	27 (24–31)
Hypertension	71%
Chronic kidney disease	23%
Coronary artery disease	30%
NYHA	
class I class II class III class IV	26% 50% 22% 2%
*Haemodynamic variables*	
Heart rate, beats per minute	71 (60–81)
Systolic blood pressure, mmHg	128 (111–143)
Diastolic blood pressure, mmHg	76 (67–86)
Elevated filling pressure	79 (53%)
PCWP, mmHg	17 (12–20)
LVEDP, mmHg	14 (10–21)
Mean PLA, mmHg	15 (10–18)

PCWP, Pulmonary capillary wedge pressure; LVEDP, Left ventricular end-diastolic filling pressure; PLA, Direct left atrial pressure

The total study population consisted of 148 patients, and 74% of patients were in heart failure NYHA class ≥ 2. Median LV EF was 55%, and 67% had LV EF ≥ 50%. Median heart rate was 71 (IQR: 60–81) beats per minute, indicating good rate control.

**Fig. 1:**
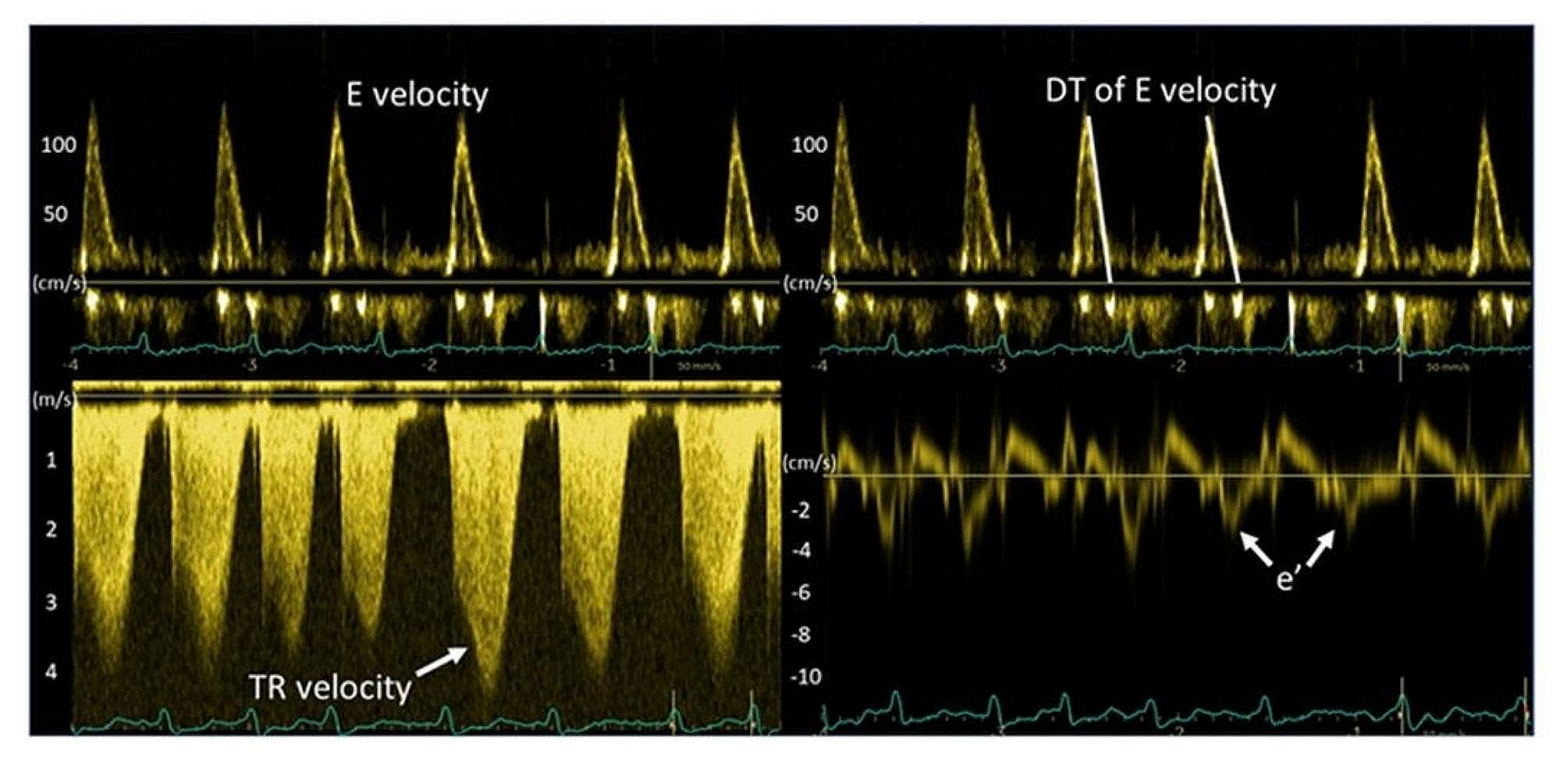
Echocardiographic recordings in a patient with elevated left ventricular filling pressure: Mitral E velocity of 120 cm/s, deceleration time of mitral E of 120 ms, peak TR velocity of 4.2 m/s, septal e’ of 3 cm/s and septal E/e’ of 40; all consistent with elevated LV filling pressure. Pulmonary capillary wedge pressure was 25 mmHg

The LV filling pressure was normal (< 15 mmHg) in 69 (47%) patients, while 40 (27%) patients had slightly elevated LV filling pressure (15–19 mmHg) and 39 (26%) had markedly elevated LV filling pressure (≥ 20 mmHg). The LV filling pressure was elevated in 43% of patients with reduced LV EF and in 52% of patients with preserved LV EF. Figure 1 shows representative recordings of key echocardiographic parameters.

### Correlation with LV filling pressure

Table 2 shows data on each of the echocardiographic parameters.

**Table 2:** Availability of echocardiographic parameters, their values, and the correlation to LV filling pressure

	All patients			LV EF < 50%			LV EF ≥ 50%		
Variables	n	Median (IQR)	r-value	n	Median (IQR)	r-value	n	Median (IQR)	r-value
LVEDV, ml	107	125 (84–160)	0.16	46	153 (119–199)	0.10	60	112 (82–145)	0.23
LVESV, ml	106	59 (39–97)	0.22*	46	98 (65–139)	0.21	59	43 (31–62)	0.28*
LV mass index, g/m^2^	141	107 (88–134)	0.16	44	134 (106–163)	0.03	91	99 (81–117)	0.22*
LA reservoir strain, %	139	10 (7–14)	0.21*	43	9 (5–13)	0.30*	93	10 (8–14)	0.16
LAVImax, ml/m^2^	145	52 (41–70)	0.06	48	57 (40–75)	0.06	97	50 (42–69)	0.05
LAVImin ml/m^2^	109	41(29–53)	0.10	47	42 (31–53)	0.14	62	39 (29–51)	0.04
LV Ejection fraction, %	146	55 (43–63)	0.18*	49	36 (28–43)	0.46*	97	58 (55–66)	0.03
Mitral E velocity, cm/s	145	86 (75–102)	0.27*	44	82 (71–107)	0.38*	94	87 (75–100)	0.19
DT of mitral E, ms	144	160 (130–182)	0.07	43	169 (136–192)	0.41*	94	155 (126–180)	0.05
PV S velocity, cm/s	62	31 (20–43)	0.10	26	28 (20–39)	0.21	33	33 (24–41)	0.08
PV D velocity, cm/s	61	55 (43–66)	0.32*	26	56 (41–69)	0.50*	33	55 (46–66)	0.16
PV S/D ratio	61	0.53 (0.38–0.80)	0.33*	26	0.54 (0.33–0.87)	0.40*	33	0.53 (0.41–0.71)	0.21
DT of PV D velocity, ms	80	234 (176–273)	0.01	17	256 (236–326)	0.10	63	223 (170–269)	0.04
Septal e’, cm/s	143	7.2 (5.8–9.1)	0.09	44	5.7 (4.3–7.3)	0.07	92	8.0 (6.4–9.2)	0.04
Lateral e’, cm/s	142	10.0 (7.8–12.8)	0.08	44	8.7 (5.9–10.0)	0.01	91	10.8 (8.3–13.1)	0.09
Septal s’, cm/s	114	5.4 (4.7–6.1)	0.11	36	4.9 (3.0–6.0)	0.21	71	5.6 (5.0-6.2)	0.06
Lateral s’. cm/s	81	7.0 (5.0–8.0)	0.17	35	5.0 (4.3–7.8)	0.20	39	7.0 (6.0–9.0)	0.19
Average E/e’	141	11 (8–14)	0.28*	43	13 (10–18)	0.33*	91	10 (8–12)	0.22*
Septal E/e’	142	12 (9–16)	0.28*	43	16 (11–21)	0.37*	92	11 (9–14)	0.18
TR velocity, m/s	129	2.5 (2.3–2.9)	0.34*	43	2.5 (2.2-3.0)	0.29	82	2.5 (2.3–2.8)	0.35*

IQR, interquartile range; LVEDV, Left ventricular end-diastolic volume; LVESV, Left ventricular end-systolic volume; LV, Left ventricular; LA, left atrial; LAVI, Left atrial volume index; DT, Deceleration time; PV, Pulmonary vein; S, Systolic; D, Diastolic; TR, Tricuspid regurgitation. *indicates a statistically significant correlation, where *p* < 0.05. Note that the number of patients with LV EF < 50% and LV EF LV EF ≥ 50% do not necessarily add up to all patients as LV EF measurement was not available in all cases

**Fig. 2:**
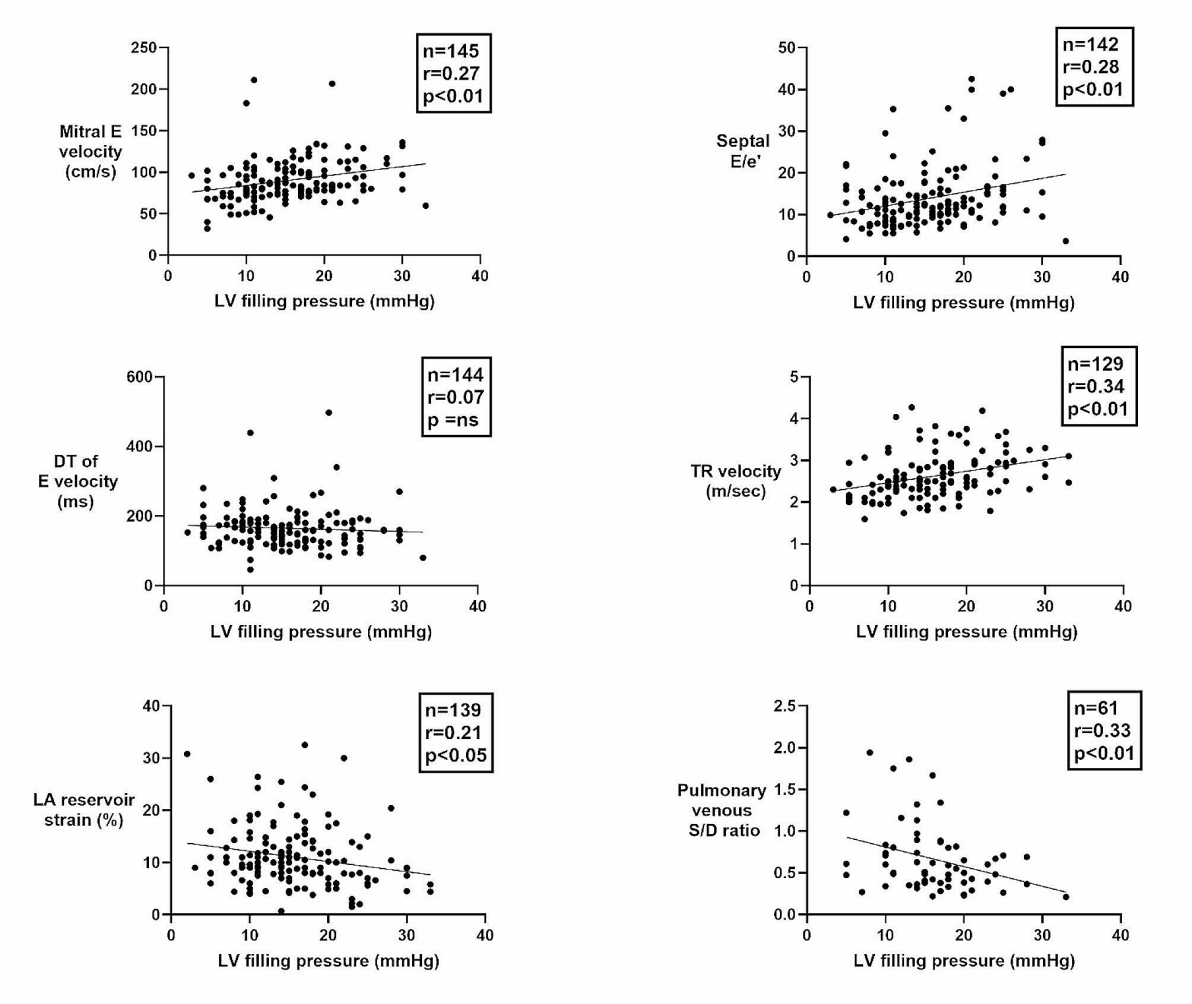
Regression plots showing association between echocardiographic parameters and LV filling pressure. BMI, Body mass index; E, early-diastolic mitral flow velocity; e’, mitral annular velocity; DT, deceleration time; LA, left atrial; LV, left ventricular; TR, Tricuspid regurgitation

The following parameters showed significant correlation with LV filling pressure in the univariate analyses; Mitral E velocity, PV diastolic velocity, PV systolic/diastolic velocity ratio (S/D ratio), TR velocity, septal E/e’ and average E/e’, LA reservoir strain, LV end-systolic volume, and LV EF. Figure 2 shows associations between echocardiographic parameters and LV filling pressure.

**Fig. 3:**
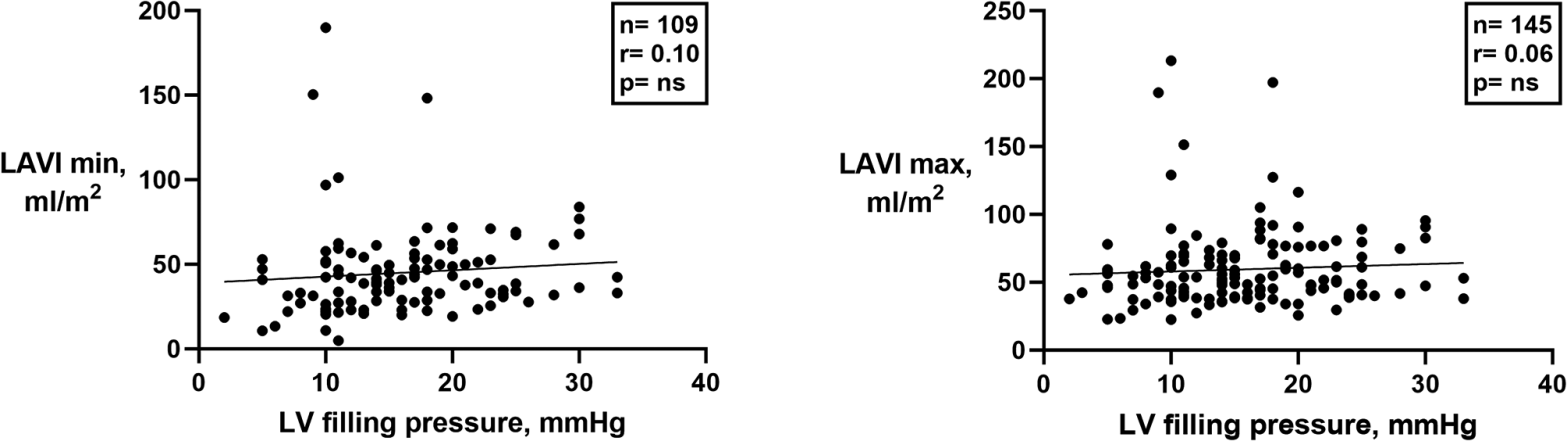
Regression plot of left atrial volumes versus LV filling pressure. Left atrial minimum (left panel) and maximum (right panel) volume indices showed no correlation to LV filling pressure. LV, left ventricular; LAVI, left atrial volume index

As shown in Fig. 3, neither maximum nor minimum left atrial volume index (LAVI) correlated significantly with LV filling pressure.

**Fig. 4:**
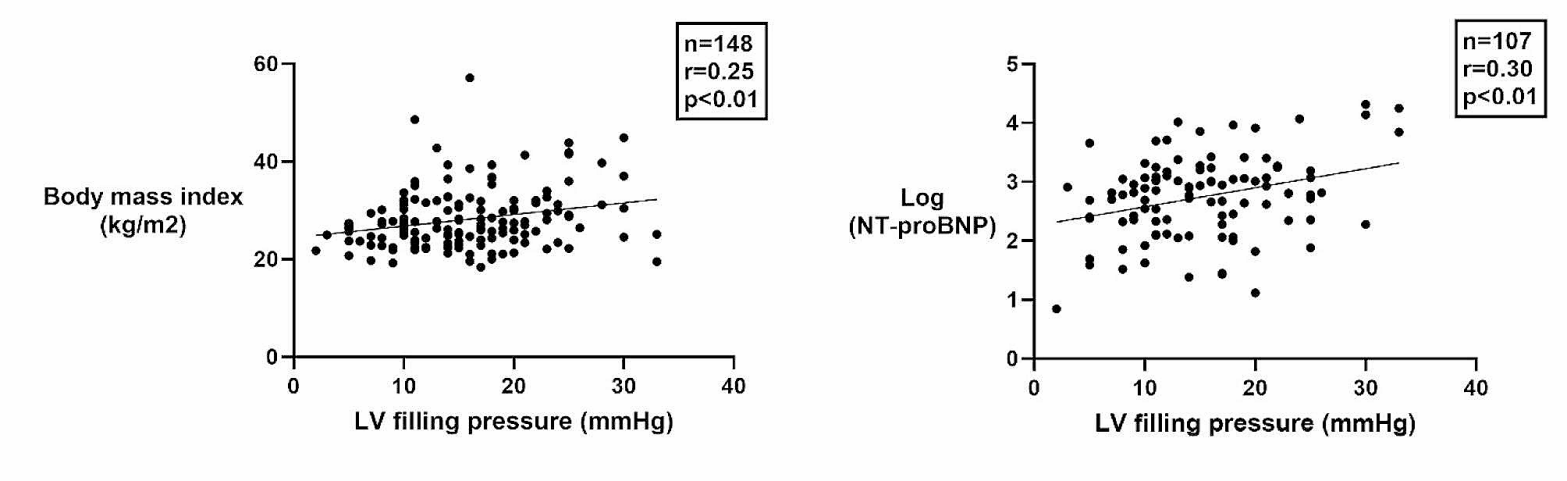
Regression plot of body mass index (BMI) and NT-proBNP versus LV filling pressure. Logarithmic scale of NT-proBNP showing moderate correlation to LV filling pressure. LV, Left ventricular

Of the clinical parameters that were studied, only body mass index (BMI) (*r* = 0.24, *p* < 0.01) showed significant correlation with LV filling pressure (Fig. 4). There was also a significant correlation between Log(NT-proBNP) and LV filling pressure (*r* = 0.30, *p* < 0.01) (Fig. 4).

### Evaluating LV filling pressure

**Fig. 5:**
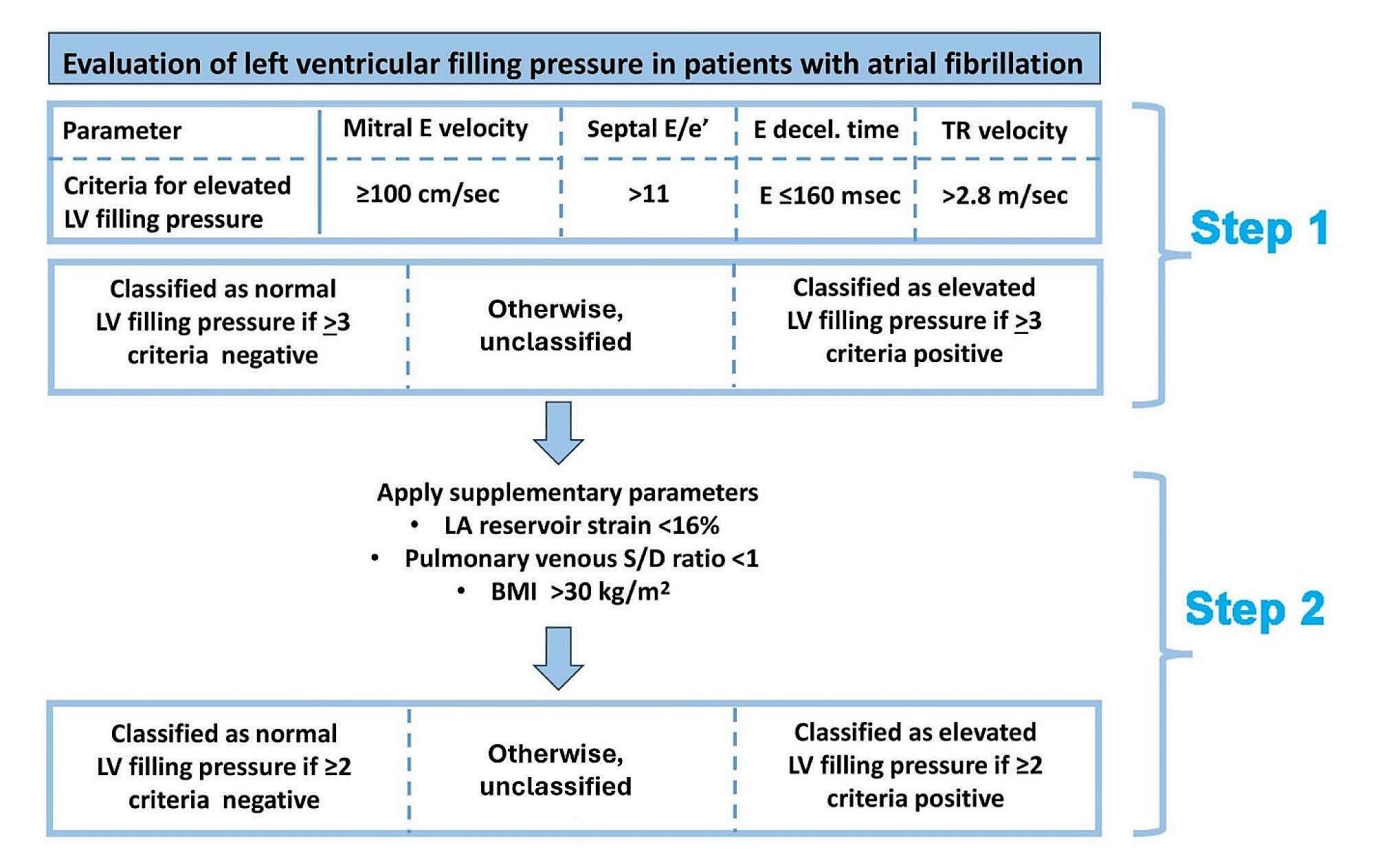
Evaluation of left ventricular filling pressure in atrial fibrillation: The first step of the algorithm consists of 4 echocardiographic parameters applied to all patients, classifying them as having normal or elevated LV filling pressure. If a patient is unclassified by the first step in the algorithm, then 3 supplementary parameters in the second step of the algorithm are applied to further classify that patient. LV, Left ventricular; E, Early diastolic; Decel, Deceleration; TR, Tricuspid regurgitation; LA, Left atrial; BMI; Body mass index

An algorithm based on multiple parameters was tested to differentiate between normal and elevated (≥ 15 mmHg) LV filling pressure (Fig. 5). The parameters in the proposed algorithm were selected based on previous literature and on which parameters had strongest associations with filling pressure in the present study.

The algorithm has two steps, as illustrated in Fig. 5. In the first step, patients are classified according to LV filling pressure as normal or elevated based on four echocardiographic parameters. Patients who remained unclassified in the first step in the algorithm were evaluated in the second step with three supplementary parameters, two from echocardiography and BMI as a clinical parameter.

The first step in the algorithm classifies LV filling pressure as either normal or elevated based on the four parameters mitral E velocity, septal E/e’, E deceleration time (DT) and TR velocity. For septal E/e’, E DT, and TR velocity, cutoff values are according to current guidelines [1]. For mitral E velocity, the cutoff value is based upon data in the present study and on observations in a large study on healthy subjects [16]. Cutoff values for each parameter are shown in Fig. 5.

In patients with at least 3 negative or 3 positive criteria, LV filling pressure was classified as being normal or elevated, respectively, and unclassified if < 3 criteria were either negative or positive. With this approach, LV filling pressure could be classified as either normal or elevated in 93 (63%) of the 148 patients, which left 55 patients (37%) unclassified.

When applying the first step in the algorithm, the accuracy to diagnose LV filling pressure as normal or elevated was 75% in the 93 patients who could be classified. The 55 patients who remained unclassified were evaluated in the second step of the algorithm.

In the second step, three supplementary parameters were used, as illustrated in Fig. 5. This included LA reservoir strain and the pulmonary venous S/D velocity ratio, both of which are established markers of LV filling pressure in patients in sinus rhythm and showed significant correlation with LV filling pressure in the present study. As a third parameter, we used BMI, which showed a statistically significant association with LV filling pressure. The cutoff values for the supplementary parameters were selected based on recommendations in existing guidelines and published reference values from studies on patients in sinus rhythm [1, 16–18]. After applying the supplementary parameters to the 55 initially unclassified patients, a classification could be made in a further 33 patients (60%), with an accuracy of 73% in the second step of the algorithm.

**Fig. 6:**
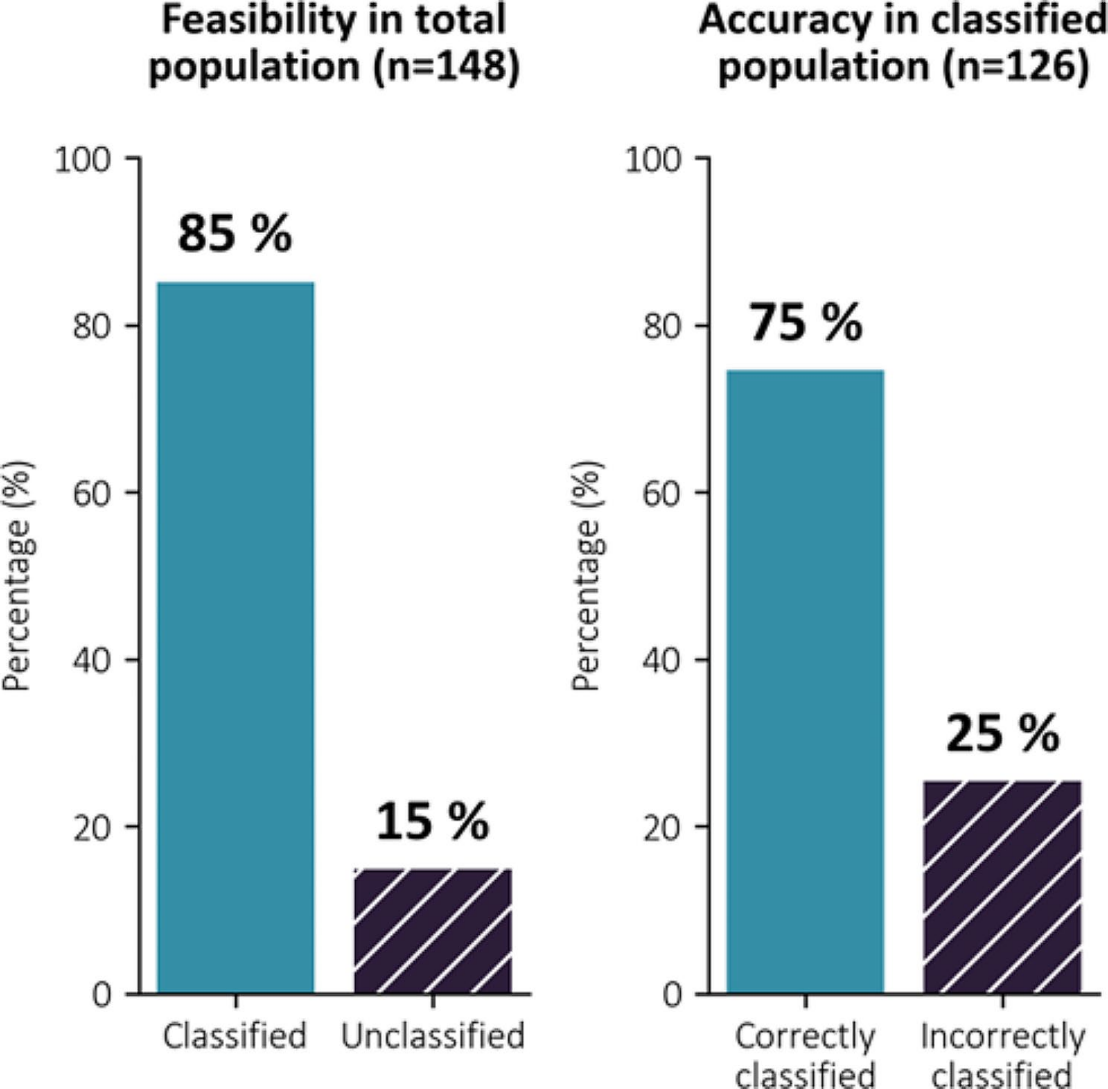
Accuracy and feasibility of the proposed algorithm. Left panel shows the feasibility of the full algorithm; 85% of patients could be classified as normal or elevated LV filling pressure. Right panel shows that the accuracy of the full algorithm applied to the patients that could be classified was 75%. LV; Left ventricular

When applying the full algorithm (both Step 1 and Step 2), a classification could be made in 126 of the 148 patients (85%), leaving 22 patients (15%) unclassified (Fig. 6). Furthermore, when applying the full algorithm, LV filling pressure was correctly diagnosed as normal or elevated in 75% of the 126 classified patients (Fig. 6).

Sensitivity and specificity of the full algorithm to diagnose elevated LV filling pressure were 74% and 76%, respectively (Table 3).

**Table 3:** Testing of the proposed algorithm (Fig. 5) for evaluation of LV filling pressure in atrial fibrillation

	Main parameters Step 1	Supplementary parameters Step 2	Full algorithm
Sensitivity	67%	90%	74%
Specificity	84%	46%	76%
PPV	82%	72%	78%
NPV	70%	75%	71%
AUC	0.76	0.68	0.75
Accuracy	75%	73%	75%

PPV: Positive predictive value; NPV: Negative predictive value; AUC: Area under curve

There was no significant difference in algorithm performance when comparing the groups with preserved and reduced LV EF. The accuracies were 75% and 76%, respectively (Table 4).

**Table 4:** Performance of the proposed algorithm in patients with normal and reduced left ventricular ejection fraction

	All patients (*n* = 148)		LV EF < 50% (*n* = 49)		LV EF ≥ 50% (*n* = 97)	
	Accuracy	Feasibility	Accuracy	Feasibility	Accuracy	Feasibility
Step 1	75%	63%	77%	67%	74%	60%
Step 2	73%	60%	70%	67%	77%	58%
Full algorithm	75%	85%	76%	89%	75%	83%

There was no significant difference in performance of the algorithm for patients referred for right heart catheterisation (PCWP measurements), compared to those studied with left heart catheterisation (direct LA pressure or LVEDP), 74% vs. 75% accuracy.

## Discussion

This study investigates how echocardiography can be applied to evaluate LV filling pressure in patients with atrial fibrillation. When testing several echocardiographic indices, including some that have shown good correlation with LV filling pressure in studies of single parameters, the conclusion was that no single parameter could be recommended for clinical use. However, when combining several parameters in a decision algorithm, it was possible to differentiate between patients with normal and elevated LV filling pressure with moderate accuracy (75%) and high feasibility (85%).

### Selection of markers of LV filling pressure

The proposed algorithm for evaluation of LV filling pressure has two steps. It starts with four echocardiographic parameters that are established markers of LV filling pressure in patients in sinus rhythm, and each parameter has a mechanistic rationale for reflecting LV filling pressure regardless of heart rhythm. All four parameters have high feasibility for measurement during a routine clinical study.

This set of parameters includes peak mitral E velocity, which is determined by the peak diastolic LA-to-LV pressure difference, and therefore tends to increase along with elevation of LA pressure. Another parameter included in the first step is septal E/e’ which is also related to LA pressure and LV diastolic function as it combines E with e’ as a marker of LV relaxation. Use of septal e’ rather than lateral e’, or the average of septal and lateral e’ in this ratio, is in keeping with recommendation for patients with atrial fibrillation in the ASE/EACVI guideline on evaluation of diastolic function [1].

The third parameter is mitral E DT, which is a marker of LV diastolic function since it tends to shorten when there is increased LV chamber stiffness and elevated LV diastolic pressure [19]. The fourth parameter is peak TR velocity as indicator of pulmonary artery systolic pressure, which tends increase to maintain transpulmonary flow when there is elevated left atrial pressure.

When applying these four parameters, the algorithm differentiated between normal and elevated filling pressure with an accuracy of 75%. With this approach, however, 37% of the patients could not be classified, and therefore a set of supplementary parameters were added in step 2 of the algorithm.

Several variables could have been selected as supplementary parameters to classify the indeterminates regarding LV filling pressure. When applying the three supplementary parameters LA reservoir strain, pulmonary venous S/D velocity ratio, and BMI on the initially unclassified patients, the overall feasibility to classify patients increased to 85%, while accuracy remained the same at 75% (Fig. 6).

### Comparison with previous studies and methodology issues

In previous smaller single-centre studies of atrial fibrillation, several echocardiographic parameters were shown to have a strong association with LV filling pressure [1, 11–14]. This includes septal E/e’, mitral E and its DT, isovolumic relaxation time (IVRT), and DT of the pulmonary venous D velocity. In the present study, parameters with strongest associations with LV filling pressure included septal E/e’, mitral E velocity, and peak TR velocity. The feasibility of obtaining these parameters was also very good.

In this study, we did not observe a significant association between mitral E DT and LV filling pressure. Mitral E DT, however, is recommended in patients with atrial fibrillation in the EACVI/ASE guideline for evaluation of diastolic function [1], was shown previously in two studies to be associated with LV filling pressure in atrial fibrillation [12, 13] and has high feasibility. For these reasons we decided to include it in the proposed algorithm.

Each of the three parameters in the second step of the proposed algorithm showed significant association with LV filling pressure. Whereas data on pulmonary venous S/D ratio was unavailable in a significant fraction of patients, the total feasibility for the algorithm to classify patients was high. Lack of data on pulmonary venous velocities in several patients reflects the partly retrospective design of the study.

Our algorithm for evaluation of filling pressure might have used alternative echocardiographic parameters, such as the DT of the pulmonary venous D velocity or IVRT. We observed no significant correlation with LV filling pressure for the DT of pulmonary venous D velocity. In two rather small studies, however, one in sinus rhythm patients and one in atrial fibrillation patients, the DT of the pulmonary venous D velocity was found to be associated with LV filling pressure [13, 20]. The potential of this parameter should be explored in future studies on evaluation of LV filling pressure in atrial fibrillation.

In the present study, IVRT was measured in only 65% of the patients and methods for measurements varied between centres. Therefore, we decided not to include this parameter in our validation. As shown recently, values for IVRT are dependent on methodology [21], suggesting the need for standardisation when using this parameter to evaluate LV filling pressure.

Left atrial volume is an established marker of LV filling pressure in patients in sinus rhythm, but the present study showed no significant correlation with LV filling pressure for LAVImin or LAVImax in atrial fibrillation (Fig. 3). Left atrial remodelling due to chronic atrial fibrillation leads to dilatation of the LA even in the absence of elevated LV filling pressure, thereby skewing the results. NT-proBNP was associated with LV filling pressure, but scatter was substantial. Furthermore, in daily clinical practice NT-proBNP is often not available, and therefore we did not include it in our algorithm which is intended for application during echocardiographic studies.

A challenge when evaluating LV diastolic function in atrial fibrillation is the variability in heart cycle length, which leads to marked beat-to-beat variability in LV filling pressure. This may have contributed to the relatively weak associations between single echocardiographic parameters and LV filling pressure observed in the present study. Sohn et al. (1999) [11] used the average of 24 beats in their analysis and showed good correlation with LV filling pressure for septal E/e’. Temporelli et al. (1996) [12], who observed a strong association between the DT of mitral E-velocity and LV filling pressure, recorded Doppler parameters and PCWP simultaneously on selected heart cycles with a cycle length equivalent to 60 to 100 beats/min. Chirillo et al (1997) [13], who observed significant correlations between LV filling pressure and DT of pulmonary venous D and mitral E velocities, also performed measurements on selected heart cycles, which represented the average heart rate of each patient.

Taken together, as suggested by previous studies, the evaluation of diastolic function in atrial fibrillation should preferably be done using standardised measurement protocols with recordings over many beats, which may be facilitated by automated measurement tools in modern echocardiography systems. Furthermore, the selection of a series of beats with R-R intervals that represent the average heart rate would be preferable.

### Definition of elevated LV filling pressure in patients with atrial fibrillation

With onset of atrial fibrillation, there is loss of the atrial kick and therefore a reduction in LV preload, which is typically compensated by systemic venoconstriction as a fast response, followed by fluid retention as a slower regulatory mechanism. These mechanisms help to bring LV end-diastolic pressure back towards normal values, but at the cost of an elevated mean LA pressure. In patients with a stiff ventricle, the atrial kick may be substantial and often exceeds 10 mmHg [22], which would tend to accentuate the compensatory elevation in mean LA pressure that follows the onset of atrial fibrillation. Therefore, there may be a rationale for having a higher value as the criterion for normal or optimal resting LV filling pressure in atrial fibrillation compared to sinus rhythm. In the present study, we investigated how criteria for elevated LV filling pressure influenced predictions by echocardiography. When using ≥ 20 mmHg rather than ≥ 15 mmHg, however, the accuracy to differentiate between normal and elevated LV filling pressure did not improve.

In the present study, we included patients with both PCWP and LVEDP as measures of LV filling pressure. For patients in sinus rhythm, LVEDP often markedly exceeds mean LA pressure. In the absence of the atrial kick and severe mitral regurgitation or stenosis, we do not expect marked differences between PCWP and LVEDP. Mean LA pressure, which was measured in some of the patients, is shown in previous studies to be essentially similar to PCWP [23].

### Clinical implications

As observed in the present study, no single echocardiographic parameter had sufficiently strong association with LV filling pressure to be recommended for clinical use as a stand-alone marker. When using the multiparameter approach, the accuracy in differentiating between normal and elevated LV filling pressure was only moderate. Therefore, clinical signs and symptoms, should always be considered when evaluating patients with atrial fibrillation for suspected pulmonary congestion.

The algorithm proposed for evaluation of LV filling pressure includes parameters that are feasible to assess in most patients undergoing routine echocardiography. Although diagnostic accuracy of the algorithm was only moderate, it has potential to serve as one of several diagnostic markers when suspecting pulmonary congestion due to high pulmonary venous pressures. By using better standardisation of measurements to minimise beat-to-beat variability in the series of heart cycles selected for analysis, there is potential to improve the echocardiographic estimates. As suggested by Bunting et al. (2021) [24], the selection of representative beats (“index beats”) for analysis is feasible in an efficient way in clinical routine. Their suggested approach for evaluation of cardiac function in atrial fibrillation was more reproducible and quicker than traditional ways of selecting beats for analysis. Future studies should consider an approach where the echocardiographic analysis is performed on beats which best represents the average heart rate. Potentially, artificial intelligence may be helpful for the identification of the most appropriate beats to analyse when evaluating LV filling pressure.

### Limitations of the study

In addition to the issues discussed above, the present study is limited by a partly retrospective design and moderate sample size. It can be argued that the proposed algorithm, which requires measurement of many parameters, is time consuming. The parameters in the suggested algorithm, however, are part of clinical routine in many echocardiography laboratories.

Ideally, each of the parameters in the algorithm should have been tested in a larger study with definition of optimal cut-off values. Furthermore, not all available markers of diastolic function were included in the evaluation. Therefore, we cannot exclude that an algorithm with other combinations of echocardiographic parameters could have provided similar or even better results than we obtained in this study. When deciding which parameters to include in the algorithm for evaluation of LV filling pressure, we selected those which had a good physiological rationale, and with demonstrated association with LV filling pressure in previous studies in sinus rhythm or atrial fibrillation. Furthermore, most of the parameters used in the algorithm showed significant association with LV filling pressure when tested as single parameters in the present study. Feasibility of obtaining the parameters in a routine clinical examination was also of importance.

## Conclusions

The present study tested the use of multiple echocardiographic parameters as indicators of LV filling pressure in atrial fibrillation, but no single parameter provided sufficient accuracy to be recommended as a stand-alone index. An algorithm that combined the best echocardiographic parameters in two steps, plus inclusion of BMI, differentiated between patients with normal and elevated LV filling pressure with moderate accuracy and high feasibility. Artificial intelligence has the potential to improve assessment of LV filling pressure and aid in developing more accurate algorithms, particularly if a greater number of patients can be included in future studies.

## Data Availability

The datasets generated and/or analysed during the current study are not publicly available due to limitations from the ethics committees in order to protect sensitive personal data.

## Abbreviations

HF: Heart failure
LV: Left ventricle/ventricular
EACVI: European Association of Cardiovascular Imaging
ASE: American Society of Echocardiography
LA: Left atrium/atrial
EF: Ejection fraction
PV: Pulmonary venous
TR: Tricuspid regurgitation
PCWP: Pulmonary capillary wedge pressure
LVEDP: Left ventricular end-diastolic pressure
BMI: Body mass index
LAVI: Left atrial volume index
DT: Deceleration time
IVRT: Isovolumetric relaxation time
